# Ethnobotany genomics - discovery and innovation in a new era of exploratory research

**DOI:** 10.1186/1746-4269-6-2

**Published:** 2010-01-26

**Authors:** Steven G Newmaster, Subramanyam Ragupathy

**Affiliations:** 1 Botany Division, Centre for Biodiversity Genomics and Biodiversity Institute of Ontario Herbarium, University of Guelph, Guelph, Ontario, Canada

## Abstract

We present here the first use of DNA barcoding in a new approach to ethnobotany we coined "ethnobotany genomics". This new approach is founded on the concept of 'assemblage' of biodiversity knowledge, which includes a coming together of different ways of knowing and valorizing species variation in a novel approach seeking to add value to both traditional knowledge (TK) and scientific knowledge (SK). We employed contemporary genomic technology, DNA barcoding, as an important tool for identifying cryptic species, which were already recognized ethnotaxa using the TK classification systems of local cultures in the Velliangiri Hills of India. This research is based on several case studies in our lab, which define an approach to that is poised to evolve quickly with the advent of new ideas and technology. Our results show that DNA barcoding validated several new cryptic plant species to science that were previously recognized by TK classifications of the Irulas and Malasars, and were lumped using SK classification. The contribution of the local aboriginal knowledge concerning plant diversity and utility in India is considerable; our study presents new ethnomedicine to science. Ethnobotany genomics can also be used to determine the distribution of rare species and their ecological requirements, including traditional ecological knowledge so that conservation strategies can be implemented. This is aligned with the Convention on Biological Diversity that was signed by over 150 nations, and thus the world's complex array of human-natural-technological relationships has effectively been re-organized.

## Introduction

Ethnobotany genomics is a novel approach that is poised to lead botanical discoveries and innovations in a new era of exploratory research. The concept for this new approach is founded on the concept of 'assemblage' of biodiversity knowledge, which includes a coming together of different ways of knowing and valorizing species variation in a novel approach seeking to add value to both traditional knowledge (TK) and scientific knowledge (SK). Ethnobotany genomics draws on an ancient body of knowledge concerning the variation in the biological diversity that surrounds different cultures; combined with modern genomic tools such as DNA barcoding it also explores the natural genetic variation found among organisms. This genomic variation is explored along a gradient of variation in which any organism inhabits. We present here the first introduction to ethnobotany genomics including some background and several case studies in our lab, which define an approach to this new discipline that may evolve quickly with new ideas and technology. The motivation for this new approach is a quest to understand how the diversity of life that surrounds us can serve society-at-large with nutrition, medicine and more.

Ethnobotany implicitly embodies the concept of interdisciplinary research. The term "ethnobotany" is derived from ethnology (study of culture) and botany (study of plants); it is the scientific study of the relationships that exist between people and plants. Historically, ethnobotanists documented, described and explained the complex relationships between cultures and their utility of plants. This often included how plants are used, managed and perceived across human societies as foods, medicines, cosmetics, dyes, textiles, building materials, tools, clothing or within cultural divination, rituals and religion. Much of this research assumes that TK can be imposed upon a SK classification of living things. We suggest that this is a biased approach and call for a more unified approach that includes concept of 'assemblage' [1] a coming together of different ways of knowing and valorizing biological variation. This novel approach seeks to add value to both aboriginal knowledge and modern science such as biodiversity genomics (DNA barcoding) to understanding diversity as they work together to potentially create new knowledge. Exploring the ways in which these different knowledge practices are worked together as 'useful knowledge' [2] will show how such inquiries contribute to the common aim of the protection of cultural and biological diversity [3]. An interdisciplinary approach such as this will respond to the increasing urgent global imperatives to conserve both cultural and biological diversity as urged by the Convention of Biological Diversity [4], UNESCO's 'Man and Biosphere Programme' and the Declaration on the Rights of Indigenous People (2007).

There is a global effort to expedite the documentation and understanding of the planet's natural diversity and the scientific underpinnings of different biological classification systems [5,6]. This includes studies that have documented aboriginal classification systems for plants and animals [7-10]. Our understanding of ethnobiological classification has recently advanced and is more complex that originally thought. TK often includes multiple mechanisms of classification [11,12] that goes beyond morphology and includes sensory perception, ecology and utilitarian characters [5,13-18]. This presents an impediment to utilizing these ancient classification systems for interpreting biodiversity because they are very complicated, which requires a great deal of time to fully comprehend, reconstruct and utilize.

Ethnobotany genomics engages modern tools that can overcome taxonomic impediments to exploring biodiversity. Contemporary Biodiversity Genomics includes intense sampling of organisms at different taxonomic levels for the same genomic region (DNA barcode) [19]. This provides a link between variation in taxa, sequence evolution and genomic structure and function, providing a good estimate of the evolutionary process. The approach integrates "Genomic Thinking" (high-volume, high-throughput) with the natural variation encountered in ecosystems to explore biological diversity. The recent development and application of DNA-based approaches enables biodiversity genomics and the development of new areas of research such as ethnobotany genomics.

DNA barcoding is a critical technique employed in biodiversity genomics. Hebert *et al*. [19] developed DNA barcoding as a method of species identification and recognition in animals using specific regions of DNA sequence data [20]. He has developed barcoding in animals, which is well documented and can be reviewed online via the Canadian Barcode of Life [21] and the Consortium for the Barcode of Life [22]. Although the difficulties of plant barcoding have been debated [23-26], detailed studies [27-37] have demonstrated the utility of barcoding as an effective tool for plant identification. Recently DNA barcoding has been used as a modern genomics tool for identifying cryptic plant species [28-30,33,34]. The applications to Ethnobiology are discussed for the first time in the literature in this paper.

The goal of this paper is to introduce a unified approach to exploring biodiversity that draws on different knowledge systems. These systems include both traditional knowledge (TK) and scientific knowledge (SK). The later utilizes DNA barcoding, as a modern identification technique to assess inter/intraspecific genetic variation among taxa, all of which is in-trenched in alpha taxonomy. We use two case studies (Ethnobotany genomics of *Biophytum *and *Tripogon*) to present this approach as examples that other research labs might model, contributing to the assemblage of a larger body biodiversity knowledge, which includes TK and SK and perhaps creates new knowledge in the process.

## Materials and methods

### Study Area

The study site (longitude 6° 40' to 7° 10' E and latitude 10° 55' to 11° 10' N) is located within the Velliangiri holy hills, which forms a major range in the Western Ghats in the Nilgiri Biosphere Reserve. The research was conducted among seven hills with altitudes ranging from 520 m - 1840 m, which is bordered by the Palghat district of Kerala on the western boundary, the plains of Coimbatore district to the east, the Nilgiri mountains to the north, and the Siruvani hills on the southern boundary.

### Ethnobotany Surveys

**Figure 1:**
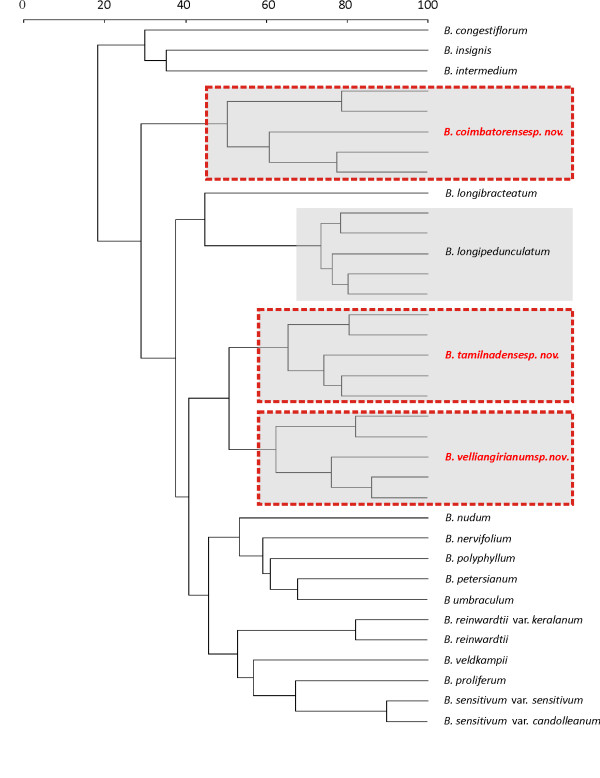
**Classification tree from DNA barcoding sequence data (rbcL, matK and trnH-psbA + 41 quantitative variables) of 19 *Biophytum *species and varieties including three new species (dotted boxes; grey boxes outlines intraspecific variation recognized as ethnotaxa)**.

**Figure 2:**
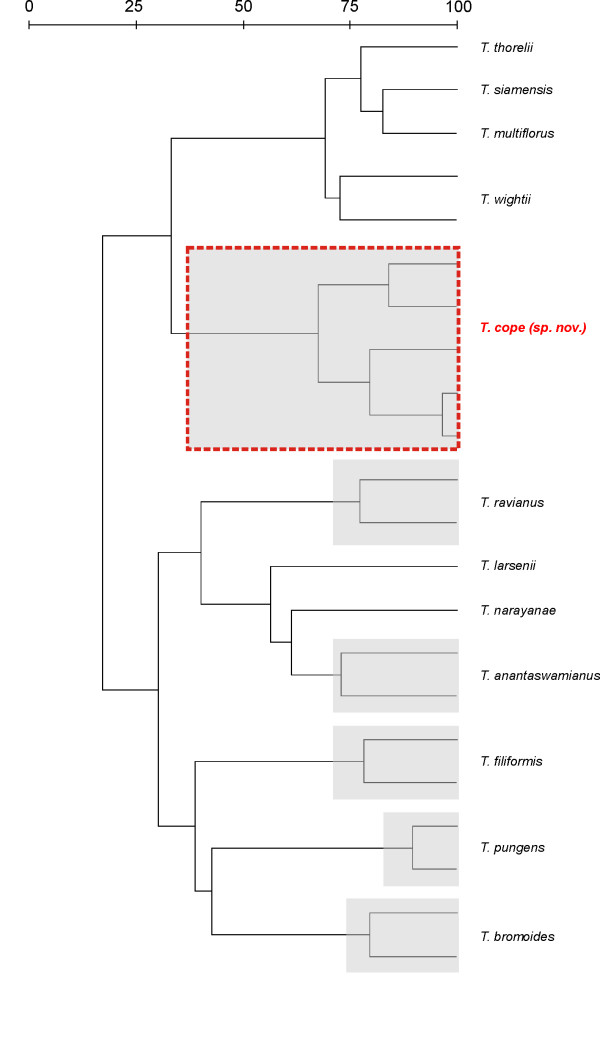
**Classification tree from DNA barcoding sequence data (rbcL, matK and trnH-psbA) of 12 *Tripogon *species one new species (dotted boxes; grey boxes outlines intraspecific variation recognized as ethnotaxa)**.

**Figure 3:**
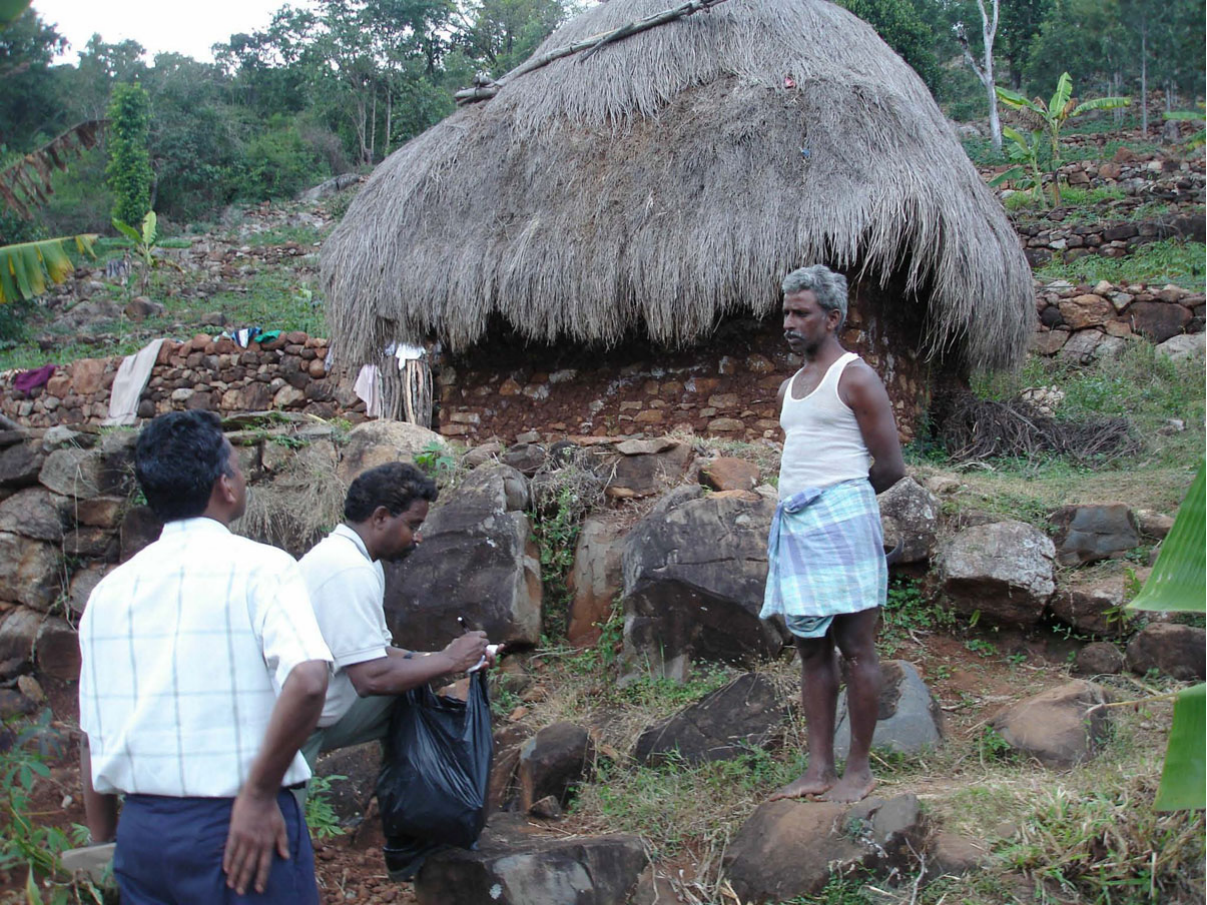
**Conducting survey with informant, Vadaman Chakkan Palanisamy**.

Floristic explorations were made within respective study areas within India [18,29,33,38-41]. Collections were made from April 2004-January 2009 and included all seasons in order to collect any ephemerals or specialized phenotypes. Six collections or "specimens" from each population were collected, labelled with locations and collection numbers for of 19 *Biophytum *species (Figure 1) and 12 *Tripogon *species (Figure 2). Corresponding field data included details of the specimens (habit, flower colour, phenology and presence or absence of latex) and environmental variables (habitat, latitude, longitude, altitude, soil type and plant associations). Multiple populations were sampled along transects separated by 2 km in order to insure that we were collecting distinct populations and not vegetative colonies. This also accounted for local morphological variants within the different ecosites. The survey used is that of earlier methodologies [12,18,33,41] to identify local experts in traditional botanical knowledge. We interviewed over 120 informants from which we selected 80 informants. Vouchers were collected and labelled for all taxa identified (Figure 3). The data were gathered in a series of structured, semi-structured and unstructured interviews, and participatory approach regarding plant uses, identification, and nomenclature. To elucidate cultural domains and determine differences in knowledge or taxonomy among aboriginals, a cross check was made with other aboriginal respondents by using various research protocols such as free recall lists, pile sorts, and consensus analysis.

### Plant Vouchers

**Figure 4:**
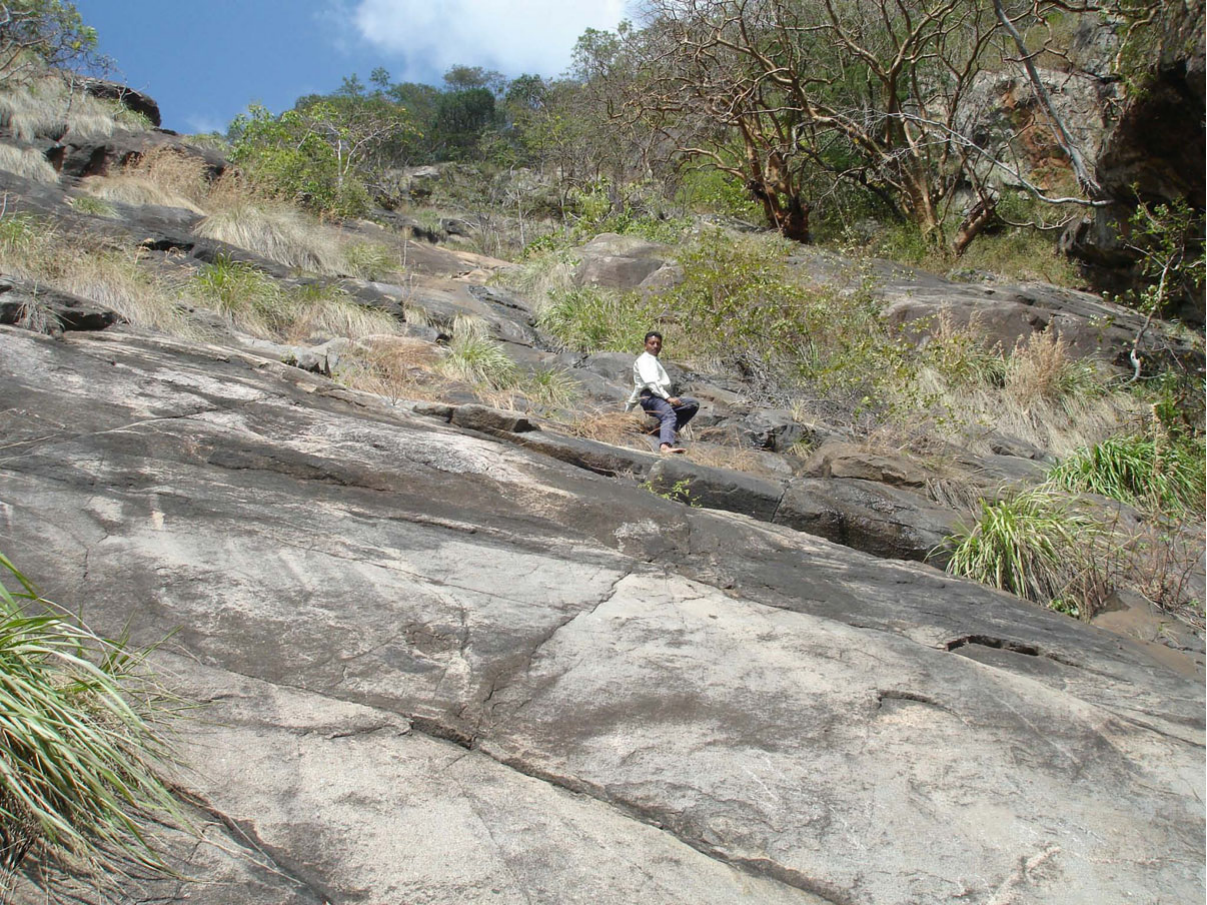
**Collecting Bare foot in the Velliangiri holy hills**.

Plant samples were collected from the aboriginal community and preserved for both herbaria and DNA barcode analysis (Figure 4). Leaf, stem and flower parts collected in situ were fixed in silica gel, FAA (50% ethanol, 5% acetic acid, 10% formalin, 35% water) and stored in 70% ethanol for morphological study ex situ. Herbarium specimens were prepared as per Jain and Rao's [42] manual and deposited in the herbarium of Kongunadu Arts and Science College, Coimbatore. The isotypes of new taxa and other taxonomically significant plant species were deposited at Madras Herbarium (MH), Southern Circle, Botanical Survey of India, Coimbatore and Ontario Agricultural College (OAC) Herbarium, Biodiversity Institute of Ontario, University of Guelph, Canada.

### Identification Analysis

Calculation of a Consensus Factor (Fic), and pile sorting relative frequency (RF) was used to test homogeneity of knowledge (SK & TK) in identifying specimens, revealing cryptic taxa or limitations of the classification without the use of molecular data. Voucher samples collected from five collection sites were systematically identified by the taxonomists and aboriginal informants. The relative frequency (RF) of each specimen from the interviews were calculated to determine a quantitative value for choosing a plant name (latin binomial or aboriginal ethno-taxon) from the pool of collected vouchers and placing it in a species concept [12]. RF is the simple calculation of the percentage of specimens associated with a taxon when taxonomists or aboriginal informants are presented with a pool of vouchers and asked to perform "pile sort". Trotter and Logan [43] provide the calculation of a Consensus Factor [Fic = Nur-Nt/(Nur-1)], which is adopted to evaluate the degree of partition into categories [44]. We have adopted this to include 'aboriginal utility' by the aboriginal informants [33,18,39,41], where Nur is the number of use-reports of informants for particular category (TK plant use) factor, where a use-report is a single record for use of a plant mentioned by an individual, and Nt refers to the number of species used for that particular category for all informants [18].

### DNA Barcoding

Three DNA regions (*rbcL*, *matK *and *trnL-F*) were selected based on the previous plant barcoding studies [27,30,35,36]. We isolated total genomic DNA from approximately 10 mg of dried leaf material from each sample using the kit, NucleoSpin^® ^96 Plant II (MACHEREY-NAGEL). Extracted DNA was stored in sterile microcentifuge tubes at -20°C. The selected loci were amplified by PCR on a PTC-100 thermocycler (Bio-Rad). DNA was amplified in 20 μl reaction mixtures containing 1 U AmpliTaq Gold Polymerase with GeneAmp 106PCR Buffer II (100 mM Tris-HCl pH 8.3, 500 mM KCl) and 2.5 mM MgCl2 (Applied Biosystems, Foster City, CA), 0.2 mM dNTPs, 0.1 mM of each primer (0.5 mM for *matK*), and 20 ng template DNA. Amplified products were sequenced in both directions with the primers used for amplification, following the protocols of the University of Guelph Genomics facility. Products from each specimen were cleaned using Sephadex columns and run on an ABI 3730 sequencer (Applied Biosystems, Foster City, CA). Bidirectional sequence reads were obtained for all the PCR products. Sequences were assembled using Sequencher 4.5 (Gene Codes Corp, Ann Arbor, MI), and aligned manually using Bioedit version 7.0.9. The sequences were used in combination with the morphometric analysis to produce classification trees.

### Morphometric Data Collection and Analyses

Morphological data variables, were recorded for all specimen collections. A matrix of specimens and morphological characters were used in a multivariate phenetic analysis. Canonical ordination was used to detect groups of specimens and to estimate the contribution of each variable to the analysis. A cluster analysis was used to classify the specimens because it is better at representing distances among similar specimens [45]. Cluster analysis was carried out using NTSYS [46]. A distance matrix was generated from the specimens and characters using an arithmetic average (UPGMA) clustering algorithm and standardized data based on average taxonomic distance subjected to the unweighted pair-group method. The resulting distance matrix from the cluster analysis used in combination with the sequence data above to produce classification trees.

## Results and Discussion

### Biophytum Ethnobotany Genomics

The genus *Biophytum *DC. (ca. 80 species, Oxalidaceae) is predominantly pantropical to subtropical in distribution [47]. *Biophytum *is one of only eight genera in three families of flowering plants (Lythraceae, Oxalidaceae, and Potederiaceae) that are tristylous [48]. The genus is poorly studied with limited floristic treatment in Knuth's [49] monograph of Oxalidaceae, which was later revised by Veldkamp [50]. The genus has been confused with that of *Oxalis*. Linnaeus described *Oxalis sensitiva *[51], from a neotype later classified as *Biophytum sensitivum *(L.) DC. Veldkamp [52] noted that the genus *Biophytum *appears to be first described in a treatise by Acosta [53], which later appeared with a plate in Clusius' treatment (1605) of Herba viva. A brief narrative of the historical nomenclature on *Biophytum *of the old world is provided by Veldkamp [52]. Veldkamp [52] states that there is no comprehensive treatment of the genus, which contains many undescribed species.

The contribution of the local aboriginal knowledge concerning variation in *Biophytum *within India is considerable. India has a high diversity of *Biophytum*; the *Biophytum *flora of India is currently represented by 17 species and two varieties of which four species are endemic, representing taxa that are in need of conservation status and protection [54]. Recent floristic surveys are reporting considerable diversity within protected religious areas in India, some of which preserve a significant portion of the *Biophytum *flora [55,18,33]. All 19 species and varieties of *Biophytum *in this study are found in the Western Ghats, which is part of Nilgiri Biosphere Reserve (NBR) in Tamil nadu. The Velliangiri hills of India are also known for their rich anthropogenic diversity. The aboriginals living in the Velliangiri hills are the "Malasars, Mudhuvars and Irulas" [11,12,18,33,41]. They have accumulated extensive ethnobotanical knowledge by their long association with their diverse, local flora [38]. In our floristic study within the Velliangiri hills we recorded 177 plants, which are used by the local people for various purposes [12,18]. These aboriginals recognize plants of the genus *Biophytum *("thottal sinungi", trnsl. 'touch me not') naming and identifying many ethnotaxa including an ecological knowledge of them [11,12,38]. It is this TK that provided clues to the identity of several new species [29,55] while working with the aboriginals in the Velliangiri hills. The respective classifications of the genus *Biophytum *using both SK and TK are not homogeneous. Taxonomists identified taxa with 84% (RF) accuracy, while the Aboriginal informants identified the same specimens with 97% (RF) accuracy [38]. Consensus factors were high (Fic = .94-.99) and not partitioned among the Aboriginal informants. The TK classification recognizes considerable fine scale variation among *Biophytum *samples (Figure 1). The TK classification of *Biophytum *is hierarchical, employing several TK classification characters; morphology, ecology, experience, gestalt and utility including 4 secondary classification mechanisms (e.g., nutritional, medicinal, technical or ritual). Interestingly these new species corresponded to unique aboriginal taxa with respective nomenclature and medicinal use [29,30,12,33,18,41].

DNA barcoding validated three new cryptic species to science that were previously recognized by TK classifications of the Irulas and Malasars. These species include 1) 'Vishamuruchi' (translation - detoxification of the poison; *Biophytum coimbatorense *sp. nov.), which is used as an antidote for poisonous scorpion bites, 2) 'Thear chedi' (translation - Chariot umbrella; *Biophytum tamilnadense *sp. nov) is used as a bait plant for fish and crab and 3) 'Idduki poondu' (translation - between the rock; *Biophytum velliangirianum *sp. nov.) is used for curing ear aches. A Classification tree from DNA barcoding sequence data (*rbcL*, *matK *and *trnH-psbA *+ 41 quantitative variables) resolved 19 *Biophytum *species and varieties including the three new species (Figure 1). DNA barcoding discriminated the cryptic ethnotaxa *Biophytum coimbatorense *sp. nov. ('Vishamuruchi') from the morphologically similar species of *B. longipedunculatum *Govind. ('Thotal sinungi'). Amplifications were highly specific with a clear background in the agarose gel. Although there were no differences in the *rbcL *or *atpF *sequences for these two cryptic species, the *matK *and more variable non-coding spacer regions such as *trnH-psbA *sequences were consistently different. Several segregating sites in the *matK *sequences are found consistently among the five distant populations. Several other studies [30,36] have also found that closely related species are not distinguished by several plastid regions like *rbcL *or *atpF*.

Ethnobotany genomics is currently being used to determine the distribution of rare species and their ecological requirements, including traditional ecological knowledge so that conservation strategies can be implemented. We are currently conducting further research on more species in the genus *Biophytum *in collaboration with several other aboriginal cultures in order to resolve species concepts within the world distribution and provide a phylogeny for the genus. Combined with a further biological and ecological data this information will contribute to conservation initiatives at a global scale.

### *Tripogon* Ethnobotany Genomics

The genus *Tripogon *Roem. & Schult. consists of nearly 40 species in tropical and subtropical regions [56-58]. The diversity of this genus of grass has been described thoroughly within the catalogue of world grasses by Peterson *et al*. [59], a revision of African species of *Tripogon *[60,61], the description of new species of *Tripogon* from Africa [62], a summary of grass genera worldwide [56], an online world grass flora by [58], and nomenclature changes by Veldkamp [63]. Rúgolo de Agrasar & Vega [64] reported that Indo-Asia constitutes the centre of diversity for this genus, with 23 species of which 16 species are native to China and 21 species including eight endemics are native to India [29]. Most of what has been published within the Indian flora and includes three new species of *Tripogon*[65-67,29].

We recently discovered a new species of *Tripogon *(*T. cope *Newm.) during an ethnobotany genomics study in the Nilgiri Biosphere Reserve, Western Ghats, India [29]. We worked with aboriginal informants who are members of the local hill tribes (Irulas and Malasars). The informants revealed ethno taxa that we later confirmed to be a new species. The ability of our field taxonomists and the Hill Tribe informants to identify species in the genus *Tripogon *was high, but the respective classifications of SK and TK are not homogeneous. Our taxonomists identified seven taxa from the 40 specimens with 96% (RF) accuracy among individuals. Aboriginal informants identified eight taxa from the same 40 specimens with 98% RF among the informants. A closer investigation of the voucher samples revealed that what we called *T. wightii *the informants split into two distinct ethnotaxa; 'Sunai pul' and 'Kattai pul'. The TK classification of *Tripogon *is hierarchical, employing several TK classification characters; ecology, experience, gestalt and utility including 4 secondary classification mechanisms (e.g., nutritional, medicinal, technical or ritual). An additional TK character used to distinguish 'Sunai pull' was that it is a 'hot' plant (see discussion below).

The cryptic ethnotaxa 'Sunai pul' and 'Kattai pul' have utility in the local hill tribes. Our ethnobotany surveys concluded that there was no partition of Fic among the 'Malasars and Irulas'. High consensus factors (0.95-0.99) confirmed that seven of the ethnotaxa are commonly used for a variety of purpose: snake hunting, fodder for domesticated animals and thatching. The new cryptic ethnotaxa 'Sunai pul' is a unique grass which is very important to both cultures with ritualistic and economic utility. 'Sunai pul' was not distinguished by the SK classification with vouchers lumped within the taxonomy of *Tripogon wightii*, which was labelled as 'Kattai pul' within the TK classification.

Further research validated that the cryptic ethnotaxa 'Sunai pul' was indeed a new species. Morphometric [29] and genetic studies [33] confirmed that the cryptic ethnotaxa 'Sunai pul' (*Tripogon cope *Newm.) was distinct from the morphologically similar species of *Tripogon wightii *('Kattai pul'). We looked at herbarium vouchers and found that the close resemblance of *T. cope *to *T. wightii *has resulted in misidentifications by taxonomists during previous botanical surveys. Although the hill tribes can easily identify these species, these cryptic species are only differentiated by minor floral characters; slight variation (1 mm) in the rachilla internodes and the number (1-3) of awns at the lemma apex. The local aboriginal classification systems species are clearly discriminated by different life cycles. We grew the plants in the greenhouse and found that 'Sunai pul' (*T. cope*) is an annual and 'Kattai pul' (*T. wightii*) is a perennial. We also used DNA barcoding to discriminate the new species (Fig 2). Our classification tree from DNA barcoding sequence data (*rbcL*, *matK *and *trnH-psbA*) clearly distinguished the 12 *Tripogon* known species from *T. cope *(Fig. 2). Intraspecific variation within the classification tree are recognized by the hill tribes as ethnotaxa of which 'Sunai pul' (*T. cope*) and 'Kattai pul' (*T. wightii*) are clearly differentiated. The DNA amplifications were highly specific with a clear background in the agarose gel. The *matK *and *trnH-psbA *sequences had several segregating sites in sequences that were found consistently among the distant populations. There is a gross interspecific variation (p-distance 0.00234) and no intraspecific variation among *T. cope *and *T. wightii*. Interspecific variation among all eight species ranged from (p-distance 0.002-0.003). Intraspecific p-distance was 0.00 for all regions within all eight species.

## Conclusion

Although there are many descriptive qualitative surveys of TK, few studies consider aboriginal classifications with respect to TK [12,33,18,41]. These studies have revealed novel ethnomedicine such as in Ragupathy *et al*. [39] whom discovered that cryptic ethnotaxa such as 'Modakathon' (*Cardiospermum halicacabum *- balloon vine) is part of the daily healthy life style used by several aboriginal cultures to control joint pain. In many cultures *Cardiospermum halicacabum *is harvested in backyards for both medicinal and food value. In fact, it provides an income supplement for some families from impoverished communities of third world countries. The paradox is that weed scientists have described balloon vine as a poisonous, noxious weed, which should be eradicated from the globe. Ragupathy *et al*. [18] identified several ethnotaxa of which one is a traditional cure to a common ailment, rheumatoid arthritis.

In both of the case studies we presented there is considerable TK associated with the new species to science, which are traditional ethnotaxa. 'Vishamuruchi' (*Biophytum coimbatorense *sp. nov.) is a detoxification for poisonous scorpion bites. The juice or extract of roots and rhizosphere is made into fine powder that is applied to a scorpion bite. A closely related cryptic species not differentiated by the taxonomist, 'Thotal sinungi' (translation - touch me not; *B. longipedunculatum*) is used to alleviate a soar throat; the leaves are squashed in the palms of their hands to extract the juice, which is dropped into the ear three times a day for three days with immediate results within in a few hours. 'Thear chedi' (*Biophytum tamilnadense *sp. nov) is bait plant for fish and crab. The Irulas collect fresh plants from the forest and tie them in bundles weighing about 1 kg. They bring 2-3 bundles to the pond and throw them into the water and wait. As soon as they see that fish are gathering near the bundle they throw their fishing net and harvest the catch. Later, crabs will inhabit the area around the bundle and can be gathered for food. 'Idduki poondu' (*Biophytum velliangirianum *sp. nov.) grows in small pockets at high elevations and is a remedy for ear aches. The preparation is similar to that of 'Thotal sinungi' (*B. longipedunculatum*).

The new grass species in our study has considerable utility to the hill tribes. In our study we found that the 'Malasars and Irulas' classified *Tripogon *taxa into eight ethnotaxa of which seven are used for similar utility; cattle and goat feed or thatching. However, the aboriginal informants recognized a common grass as 'Sunai pul' (*T. cope*) that is clearly differentiated by them from another grass 'Kattai pul' (*T. wightii*). The etymology for 'Kattai pul' refers to a cold, hard and stout grass that lives for many seasons and is used for cattle and goat feed as many other common grasses. 'Sunai pull' is a very special species to these cultures. The etymology for 'Sunai pull' refers to the hot, bushy, hairy snake grass that lives for only one monsoon season. While working in the field, the Irulas informants first introduced us to 'Sunai pull' with a warning. "Do not step near 'Sunai pull' because this is where the cobra seeks shelter. In fact, the local Irulas snake catchers come into the hills to catch cobras among the patches of 'Sunai pull'. They told us that 'Sunai pull' is hot, or gives of heat and that the snakes like to sleep there. Snake catching is viable part of the economy of several local villages because the demand for snake venom and skins. The extracted venom is purified, frozen and then freeze-dried to make the pure venom powder that is used by government laboratories for the production of anti-venom serum. To produce just one gram of pure cobra venom, 10 snakes are needed, while to produce the same amount of the saw-scaled viper venom the Irulas have to catch 750 snakes. A gram of the venom can cost up to $1,500 (USD) for some species of vipers. The snake skin is used to make cosmetics and industry representatives (often from export companies) come to the remote villages to buy skins from the Irulas. The importance to theses aboriginal cultures is apparent; the recognition by modern science is lagging behind because of taxonomic impediments.

DNA barcoding may provide an important tool for identifying cryptic species and validating ethnotaxa. One of the greatest utilities of barcoding is its use in overcoming taxonomic impediments; identifying cryptic materials such as unknown leaves, roots, etc. Barcoding was used in the study of nutmeg [29] to identify species in the *Myristicaceae *that are primarily separated by androecium characters in small, short-lived flowers that are only available for two weeks of the year. This study identified several crytic taxa including population level differences in *Compsoneura *associated with ecotypic differences and vicariance, suggesting several new cryptic species. DNA barcoding is a tool that ethnobiologists can employ to 1) validating ethnotaxa, 2) help overcoming hurdles of ambiguity, 3) gain credibility in science, and 4) stimulate new theory on understanding, preserving biological and cultural diversity.

We have initiated further ethnobotany genomic studies in other cultures to develop theoretically sophisticated insights concerning the encounter between 'local' and 'scientific' approaches to biodiversity knowledge. These will further contribute to a body of research on the social, cultural and political underpinnings biodiversity science; our understanding of the natural variation that surrounds us. Furthermore, the research will add to a unifying global effort to speed up the documentation (via DNA barcoding) and understanding of the planet's biodiversity, while concurrently respecting cultural heterogeneity as a vital component of biological diversity. This is aligned with the Convention on Biological Diversity [4] that was signed by over 150 nations, and thus the world's complex array of human-natural-technological relationships has effectively been re-organized.

## Consent

Written informed consent was obtained for publication of accompanying images. A copy of the written consent is available for review by the Editor-in-Chief of this journal.

## Competing interests

The authors declare that they have no competing interests.

## Authors' contributions

SGN conceived and designed the study, carried out analysis and writing of the manuscript. SR carried out the field and lab work, contributed to the study design and writing of the manuscript. both authors read and approved the final manuscript.
